# Supplementary material for: The interactome of intact mitochondria by cross-linking mass spectrometry provides evidence for coexisting respiratory supercomplexes
Source: Mol Cell Proteomics. 2017 Dec 8;17(2):216–32. doi: 10.1074/mcp.RA117.000470 (PMC5795388; doi:10.1074/mcp.RA117.000470)
Supplement: Supplemental Data [file supp_RA117.000470_133922_0_supp_23978_fzffwf.zip › spectra_annotation/mito_spectra_annotation/309-1-15-1-5-1.pdf]

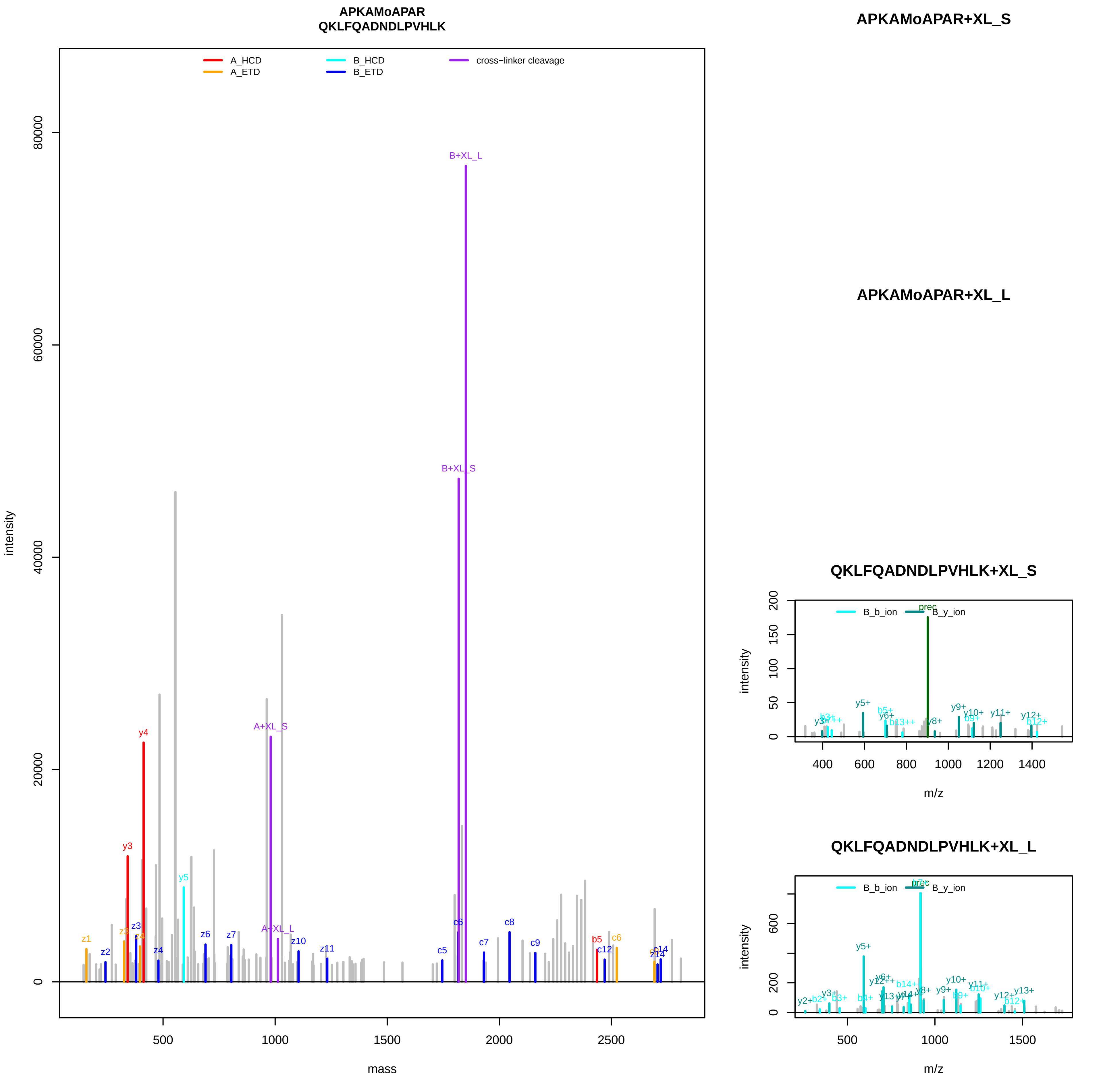
APKAMoAPAR
QKLFQADNDLPVHLK
A_HCD
A_ETD
B_HCD
B_ETD
cross−linker cleavage
B+XL_L
B+XL_S
A+XL_S
A+XL_L
y3
y4
y5
b5
z1
z2
z3
z4
z6
z7
z10
z11
z14
c5
c6
c7
c8
c9
c12
c14
intensity
mass
0
20000
40000
60000
80000
500
1000
1500
2000
2500
APKAMoAPAR+XL_S
APKAMoAPAR+XL_L
QKLFQADNDLPVHLK+XL_S
B_b_ion
B_y_ion
prec
intensity
m/z
0
50
100
150
200
400
600
800
1000
1200
1400
QKLFQADNDLPVHLK+XL_L
B_b_ion
B_y_ion
prec
intensity
m/z
0
200
600
500
1000
1500
